# Identification of transthyretin as a novel interacting partner for the δ subunit of GABA_A_ receptors

**DOI:** 10.1371/journal.pone.0210094

**Published:** 2019-01-07

**Authors:** Li Zhou, Xin Tang, Xinyi Li, Yuting Bai, Joel N. Buxbaum, Gong Chen

**Affiliations:** 1 Department of Biology, Huck Institutes of Life Sciences, The Pennsylvania State University, University Park, PA, United States of America; 2 Department of Molecular and Experimental Medicine, The Scripps Research Institute, La Jolla, CA, United States of America; Indiana University School of Medicine, UNITED STATES

## Abstract

GABA_A_ receptors (GABA_A_-Rs) play critical roles in brain development and synchronization of neural network activity. While synaptic GABA_A_-Rs can exert rapid inhibition, the extrasynaptic GABA_A_-Rs can tonically inhibit neuronal activity due to constant activation by ambient GABA. The δ subunit-containing GABA_A_-Rs are expressed abundantly in the cerebellum, hippocampus and thalamus to mediate the major tonic inhibition in the brain. While electrophysiological and pharmacological properties of the δ-GABA_A_-Rs have been well characterized, the molecular interacting partners of the δ-GABA_A_-Rs are not clearly defined. Here, using a yeast two-hybrid screening assay, we identified transthyretin (TTR) as a novel regulatory molecule for the δ-GABA_A_-Rs. Knockdown of TTR in cultured cerebellar granule neurons significantly decreased the δ receptor expression; whereas overexpressing TTR in cortical neurons increased the δ receptor expression. Electrophysiological analysis confirmed that knockdown or overexpression of TTR in cultured neurons resulted in a corresponding decrease or increase of tonic currents. Furthermore, *in vivo* analysis of TTR-/- mice revealed a significant decrease of the surface expression of the δ-GABA_A_-Rs in cerebellar granule neurons. Together, our studies identified TTR as a novel regulator of the δ-GABA_A_-Rs.

## Introduction

GABA (γ-aminobutyric acid) activates GABA_A_ and GABA_B_ receptors to mediate the majority of inhibition in the brain [1, 2]. GABA_A_ receptors (GABA_A_-Rs) are located on both synaptic and extrasynaptic membranes to mediate phasic and tonic inhibition. We have recently demonstrated that synaptic and extrasynaptic GABA_A_-Rs compete with each other to regulate the homeostasis of inhibition [3]. Deficits in GABA_A_-R-mediated neurotransmission are involved in epilepsy, anxiety, depression, schizophrenia, and autism [4–8]. Extrasynaptic GABA_A_-Rs are sensitive to steroid and alcohol regulation, and play important roles in sleep, stress, puberty, learning, and pregnancy-related mood disorders [9–11]. The δ subunit-containing GABA_A_-Rs (δ-GABA_A_-Rs) are one of the major subtypes of extrasynaptic GABA_A_-Rs, localizing in the cerebellum (α6βδ), hippocampus and thalamus (α4βδ) [9, 12–14]. Although it is well known that δ-GABA_A_-Rs mediate tonic inhibition of neuronal activity in the brain [9, 14, 15], the molecular partners that interact with the δ-GABA_A_-Rs have not yet been identified.

Here, we employed a yeast two-hybridization system to screen for δ-subunit interacting proteins from a mouse cerebellar cDNA library, a brain region in which the δ-subunit is highly expressed. We indentified transthyretin (TTR) as a novel interacting partner for the δ-subunit. Transthyretin (TTR) is a transporter for thyroxine and retinol-binding protein bound to retinol in the blood and cerebral spinal fluid [16, 17]. Plasma TTR is mainly produced by liver, while TTR in the brain is predominantly synthesized by choroid plexus [18] and by neurons under stress [19]. TTR interacts with the β-amyloid peptide (Aβ) oligomers and fibrils and may play a neuroprotective role in Alzheimer's disease [20–23].

Our current study demonstrates that TTR interacts with δ-GABA_A_-Rs and regulates their expression and function. We found that TTR co-immunoprecipitates with the δ subunit in brain lysates. Knockdown of TTR expression in cultured cerebellar granule cells significantly reduced the surface δ expression level as well as the corresponding tonic current. Conversely, overexpressing TTR in cortical neurons, which typically have low levels of δ expression, significantly increased the surface expression level of the δ subunit and the tonic current as well. Interestingly, external application of recombinant human TTR (hTTR), but not mutant monomeric hTTR, significantly increased the surface δ receptors in cultured cerebellar granule cells. We further investigated TTR regulation of the δ receptors in TTR knockout mice, and observed a significant decrease of the δ surface staining in cerebellar granule layer compared to the wild type (WT) mice. In conclusion, we discovered that TTR is a novel interacting partner for the δ-GABA_A_-Rs.

## Materials and methods

### Yeast two-hybrid assay

Mouse cerebellar RNA was extracted using Neuron spin RNA II kit (Clontech). Make Your Own “Mate & Plate™” Library (Clontech) was used to establish the mouse cDNA library from the cerebellar RNA according to the manual. Matchmaker™ Gold Yeast Two-Hybrid System (Clontech) and the established cDNA library were used in the interacting screen according to the manual. The interaction of pray (pGADT7) and bait (pGBKT7) fusion proteins were assayed by the AUR1-C, ADE2, HIS3, and MEL1 reporters. Plasmid DNA of positive clones was recovered and inserts were analyzed by sequencing.

### Plasmid constructs and transfection

The extracellular domain of the δ subunit of GABA_A_ receptor was subcloned into pGBKT7-DNA BD vector (Clontech). The full-length TTR cDNA was amplified from mouse cerebellar RNA and was cloned as BGII and EcoR fragment into pN3HA plasmid in which the GFP sequence of pEGFPC1 was replaced by triple HA (a kind gift from Dr. Yingwei Mao) or PEGFPC1 vector. The plasmid pLenti-GIII-CMV encoding the full length human TTR with RFP reporter driven by the independent promoter was purchased from Applied Biological Materials (Richmond, Canada). The GIPZ mouse TTR shRNA vector was purchased from Open Biosystem. The vectors of GABA_A_ receptor δ, α6, β3 subunits were described preciously in our publications [3, 24]. The purified proteins of human TTR and mutated monomeric hTTR are from Dr. Buxbaum' Lab (Scripps Research Institute). Primary cultured neurons were transfected using calcium-phoshphate transfection protocol as described [25]. For HEK 293T cell transfection, polyethylenimine (PEI, Polyscience) was used.

### Mice and primary neuron culture

C57BL/6 [WT (B6)] and m*ttr*-/- (mouse *ttr* knockout) mice were used similar to that described before [19]. C57BL/6 were purchased from Jackson Lab. Mice of m*ttr*-/- were from Dr. Buxbaum' Lab(Scripps Institutes). Mice of either sex were used in the experiments. Primary cerebellar and cortical tissues were dissected out from C57BL/6 mouse pups (newborn for cortical culture and postnatal 5–7 days for cerebellar culture) as described previously [3, 26, 27]. All the animal procedures were reviewed and approved by the Institutional Animal Care and Use Committee at Penn State University (IACUC# 43379). Briefly, cerebellar or cortical cells were dissociated with 0.25% Trypsin-EDTA containing 50 units/ml DNase I and then plated on a monolayer of cortical astrocytes at a density of 8,000–12,000 cells/cm^2^. The cerebellar neuron culture medium contained 500 ml MEM (Invitrogen, Eugene, OR), 10% fetal bovine serum (HyClone, Logan, UT), 10 ml B-27 supplement (Invitrogen), 100 mg NaHCO3, 20 mM KCl, 0.5 mM L-glutamine, 25 unit/ml penicillin/streptomycin, and 4 μM AraC to suppress the excessive proliferation of astrocytes. Neurons were maintained at 37°C in a 5% CO_2_-humidified incubator for 2–3 weeks. All data presented as mean ± SE. Student’s *t* test or one-way ANOVA followed with Bonferroni correction were used for statistical analyses.

### Co-immunoprecipitation and Western blot

The cerebellum tissue of adult mice was dissected out and homogenized in cold IP lysis buffer containing 25 mM Tris/HCl (pH 7.4), 150 mM NaCl, 1% NP-40, 1 mM EDTA, 5% glycerol (Thermo Scientific, Rockford, USA) with protease inhibitors and phosphatase inhibitors (Sigma), followed by incubation at 4°C for 30 min and sonication twice for 30 s each. The supernatant of brain extracts was harvested by centrifugation (12,000 g, 10 min). Protein content was measured by Bradford protein assay (Thermo Scientific). For Co-IP assay, the cerebellum extracts or the overexpressed HEK cell lysates were first pre-cleaned by incubation with Dynabeads M-280 IgG (Invitrogen) for 2 hr at 4°C. 2 μg primary antibodies of rabbit anti-TTR (DAKO), mouse anti-HA (Santacruz), or rabbit anti-myc (cell signal Tech) were added into the protein lysate and incubated overnight at 4°C, followed by adding 30 μl Dynabead M-280 anti-mouse or anti-rabbit IgG (Invitrogen) and incubating for another 4 hr at 4°C. After washing with PBS, the immunoprecipitated proteins were eluted and boiled for 10 min in NuPAGE LDS sample buffer (Invitrogen). The precipitated protein was then separated by SDS-PAGE gel and transferred to PVDF membrane. The primary antibodies used in this study included mouse anti-HA, rabbit anti-Myc, rabbit anti-GABA_A_-R δ subunit (PhosphoSolution, Aurora, USA), and mouse anti-actin (BD). Immunoblot band intensities were quantified using Image J software. All experiments were repeated at least three times independently.

### Immunocytochemistry

For live cell staining, cultured cerebellar or cotical neurons (9–12 days in vitro) were incubated in bath solution (128 mM NaCl, 30 mM glucose, 25 mM HEPES, 2 mM KCl, 2 mM CaCl_2_ and 1 mM MgCl_2_ (320 mOsm, pH 7.4) with primary anti-GABA_A_-R δ subunit or anti-transthyretin antibodies (Abcam) at 4°C for 1 hr, followed by washing with phosphate-buffered saline (PBS) and fixed for 15 min in 4% paraformaldehyde (PFA). After fixation, neurons were blocked with 5% BSA in PBS for 1 hr, followed by incubation with Dylight-conjugated anti-rabbit 546 (1:500, Jackson ImmunoResearch) or Alexa flour 488 (1:500, Molecular probes) for 2 hr under non-permeabilized condition.

For total protein staining, neurons were washed with bath solution, fixed by 4% PFA for 12 min, and followed by three times washing with PBS. Then, neurons were permeabilized with 0.2% triton in PBS for 8 min, and changed into 5% NDS + 0.1% triton in PBS for 30 min before incubating with primary antibodies in blocking buffer overnight at 4°C. After extensive washing with PBS, the coverslips with neurons were incubated with appropriate secondary antibodies for 1 hr at room temperature, and then rinsed with PBS four times. The coverslips were finally mounted with anti-fading mounting solution containing DAPI (Invitrogen). The images were taken by an epifluorescent microscope (Nikon TE 2000-S) or a confocal microscope (Olympus FV1000) and analyzed by Image J software.

For mouse brain section staining, 7–8 months old mice were anesthetized with 2.5% Avertin and then perfused with cold saline solution (0.9% NaCl) for 2 min. The whole brain was immediately taken out and cut into half and fixed in 4% PFA overnight at 4°C. The brain tissue was then cut into 45 μm slices with a vibratome (Lecia). The brain slices were washed with PBS and pretreated with 0.3% triton for 2 hr and then incubated with 5% NDS and 0.1% triton in PBS for 2 hr. The primary antibodies in blocking solution were applied to brain slices at 4°C overnight. The next day, the cells were washed with PBS and incubated with proper fluorophore-conjucated secondary antibodies for 1 hr at room temperature. After the secondary antibody incubation, the excessive antibodies were washed off with PBS and the coverslips or brain slices were mounted in anti-fading mounting solution with DAPI (Invitrogen). The images were collected on an Olympus FV1000 confocal microscope. For quantification of GABA_A_-R δ subunit signal, the mean intensity of neuronal soma was analyzed by Image J software. The colocalization was analyzed with the Intensity Correlation analysis plug-in in ImageJ (National Institutes of Health, Bethesda, MD) [28]. All experiments were repeated at least three times independently.

### Electrophysiology

TTR and TTR shRNA plasmids were transfected at 4 days in vitro (DIV) with Ca^2+^-phosphate methods described before [25]. Cover glasses with cultured cells were transferred to a recording chamber with continuous perfusion of the bath Tyrode’s solution that contains (in mM) 128 NaCl, 30 glucose, 25 HEPES, 5 KCl, 2 CaCl_2_, and 1 MgCl_2_, pH 7.3, ~320 Osm. Fire-polished borosilicate glass pipettes with resistance of 3–5 MΩ were used for recording. The internal pipette solution contained (in mM) 135 KCl, 10 HEPES, 2 EGTA, 10 Tris-phosphocreatine, 4 MgATP, and 0.5 Na_2_GTP (pH 7.3, ~300 Osm). Whole-cell recordings of THIP-induced currents were performed at room temperature in voltage-clamp mode using Multiclamp 700A amplifier (Molecular Devices, Palo Alto, CA), similar to previously described [24]. The membrane potential was held at -70 mV. Data were acquired using pClamp 9 software (Molecular Devices), sampled at 5 kHz, and filtered at 1 kHz. Cerebellar cultures were recorded around DIV 8, whereas cortical cultures were recorded around DIV 14. To examine THIP-induced currents, neurons were perfused with bath solution that contains TTX (0.5 μM) and CNQX (10 μΜ) to block voltage-gated sodium channels and AMPA/kainate receptor currents. THIP (2 μM) was then applied for 10 s through a valve-controlled drug delivery system VC-6 (Warner Instruments, Hamden, CT) to elicit stable inward current. All experiments were repeated at least three times independently.

## Results

### Transthyretin identified as the interacting partner for GABA_A_ receptor δ subunit

**Fig 1:**
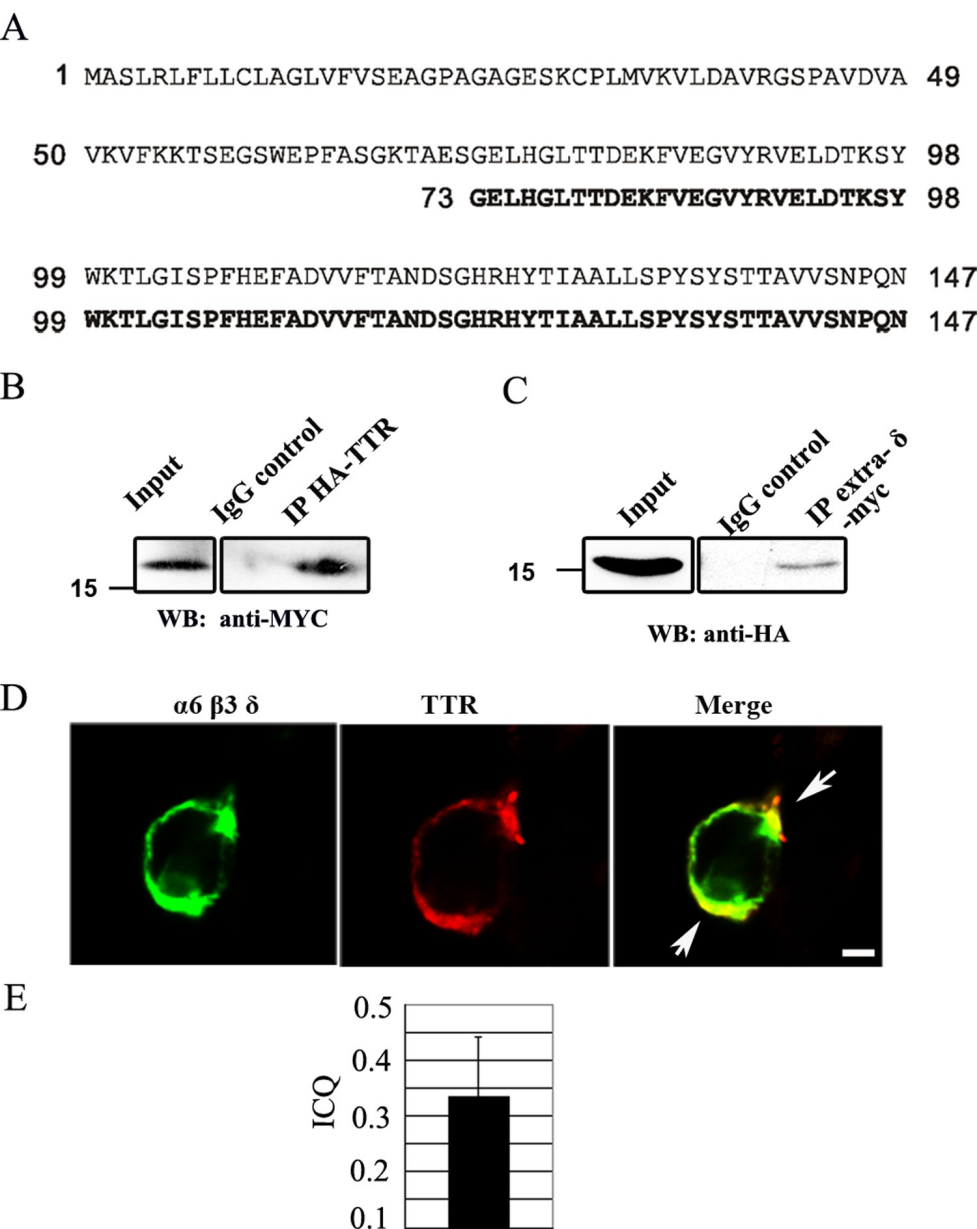
Identification of TTR as a novel interacting protein for the GABA_A_ receptor δ subunit. (**A**) Alignment of the amino-acid sequence of full length mouse transthyretin (NP_038725) with the sequence of the positive clone obtained from our yeast two-hybrid screening. (**B-C**) Co-immunoprecipitation of the HA-tagged TTR and the myc-tagged extracellular domain of the δ-subunit (extra-δ) after co-expression in HEK cells. (**D**) Confocal images showing the co-localization of the α6β3δ-receptors (green) and TTR (red) after co-expressed in HEK cells. The arrows show the coloclization sites. Scale bar, 10 μm. **(E)** The quantification of colocalization of the transfected TTR and α6β3δ receptors in HEK cell by intensity correlation analysis in Image J. The mean intensity correlation quotient(ICQ) is between 0 and +05, which means they are dependent staining.

The structure of GABA_A_-R δ subunit includes one extracellular domain (extra-δ), one cytoplasmic domain and four transmembrane domains. The N-terminus of GABA_A_-R subunit is responsible for oligomerization and receptor assembly [1]. While δ-GABA_A_-Rs have been extensively studied in terms of function and subcellular localization, the molecular partners interacting with δ-GABA_A_-Rs have not yet been identified. To understand the molecular mechanism of δ-receptor regulation, we used the extracellular domain of the δ subunit (amino acid 17–248) as the bait in a yeast two-hybrid screen to identify interacting proteins. Since the cerebellum has a high level of δ subunit expression, we made the cDNA library from adult mouse cerebellum. From this library, one of the positive clones encoding amino acid 73–147 of transthyretin (TTR) was isolated (Fig 1A). To confirm the interaction between TTR and the extra-δ in mammalian systems, HA-tagged full length TTR and Myc-tagged extra-δ were co-expressed in HEK cells, and the lysate was immunoprecipitated with HA or myc antibodies. Both HA-TTR and myc-extra-δ were co-precipitated with each other (Fig 1B and 1C). Consistently, confocal images showed that TTR was co-localized with α6β3δ receptors when co-expressed in HEK cells (Fig 1D). The co-localozation of the transfected TTR and α6β3δ receptors was analyzed using intensity correlation analysis. The mean intensity correlation quotient (ICQ) number of the co-localozation of the transfected TTR and delta subunits is 0.333±0.108 (Fig 1E), which means that the staining of TTR and α6β3δ receptors are dependent staining. Therefore, our results identified TTR as a novel interacting protein with the δ subunit of GABA_A_ receptors.

### Endogenous transthyretin interacts with δ-GABA_A_ receptors in the cerebellum

**Fig 2:**
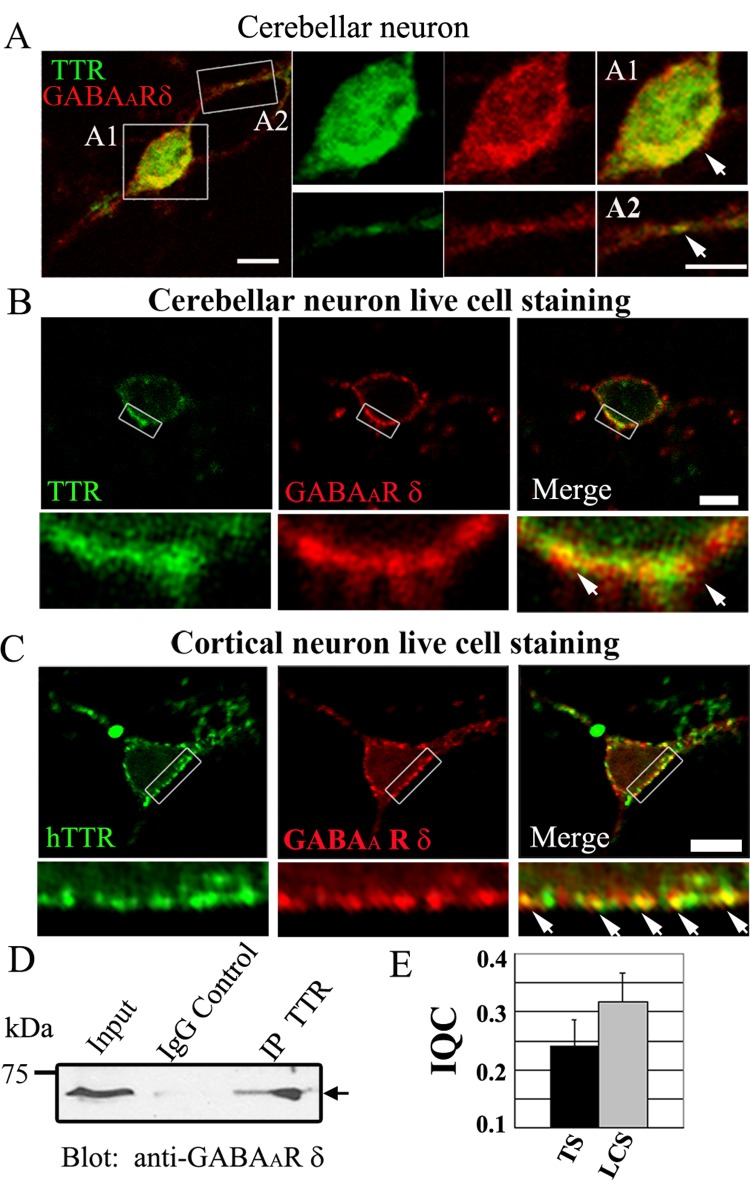
TTR interacts with the GABA_A_ receptor δ subunit *in vivo*. (**A**) Total staining of endogenous GABA_A_ receptor δ subunit (red) and TTR (green) in cerebellar granule cells in culture. Boxed areas were enlarged for better view of the colocalization. The arrows point the coloclization sites. Scale bar: 5 μm. (**B**) Live cell staining of the endogenous surface level of TTR (green) and the GABA_A_ receptor δ subunit (red) in cultured cerebellar granule cells. The arrows point the coloclization sites. Scale bar: 5 μm. (**C**) Live cell staining of cortical neurons overexpressed with hTTR (green) and rat δ subunit (red), revealing partial colocalization of the two signals. The arrows point the coloclization sites. Scale bar: 10 μm. (**D**) Co-immunoprecipitation of TTR and δ-receptors from mouse cerebellum extract. Brain lysate was immunoprecipitated with sheep anti-TTR antibody or normal sheep IgG as a control, and then immunoblotted with rabbit anti-δ antibody. **(E)** The quantification ofcolocalization of the TTR and δ-α6β3δ receptors in cerebellar neurons by intensity correlation analysis. The intensity correlation quotient(IQC) was shown for both total staining (TS) and live cell staining(LCS).

It is well know that δ-subunit-containing GABAA receptors mediate tonic inhibition in cerebellar granule cells [29], the dentate gyrus granule cells [29], alamic neurons [30], and in pyramidal neurons[31]. And recently, α1δ-subunit assemblies were shown to be present in the hippocampal interneurons [32]. These inspire us to examine the interaction between TTR and the δ subunit of GABA_A_-Rs in primary neuronal cultures. We started with an investigation of the endogenous signal of TTR and the δ subunit in cerebellar granule cells in primary cultures. After fixation and membrane permeabilization, we found that TTR and the δ subunit partially co-localized in granule cells (Fig 2A). We further performed live cell staining without membrane permeabilization and demonstrated that TTR and the δ subunit also partially co-localized on cell surface (Fig 2B). We next overexpressed human TTR (hTTR) and the rat δ subunit in cultured cortical neurons, which usually lack endogenous δ subunit, and performed live cell staining. Again, the expressed hTTR and the δ subunit were found partially co-localized in cortical neurons (Fig 2C). The quantification of both total staining and surface staining of TTR and δ-GABA_A_-Rs in cerebellar neurons was analyzed by the intensity correlation analysis. The mean intensity correlation quotient (ICQ) of total staining is 0.242±0.043, and the mean ICQ of surface staining is 0.318±0.048 (Fig 2E). The number of ICQ between 0 and +0.5 indicates that they are dependent staining. To further understand whether TTR interacts with the δ-GABA_A_-Rs in mouse brain *in vivo*, we used TTR antibodies to immunoprecipitate TTR from cerebellum lysate, which is known to contain a high level of the δ-GABA_A_-Rs [33]. Immunoblotting with the δ-specific antibodies clearly showed that TTR and the δ-GABA_A_-Rs were co-immunoprecipitated with each other in the cerebellum tissue (Fig 2D). Therefore, TTR can interact with the δ-GABA_A_-Rs in the brain in vivo.

### TTR regulates the expression of δ-GABA_A_ receptors

**Fig 3:**
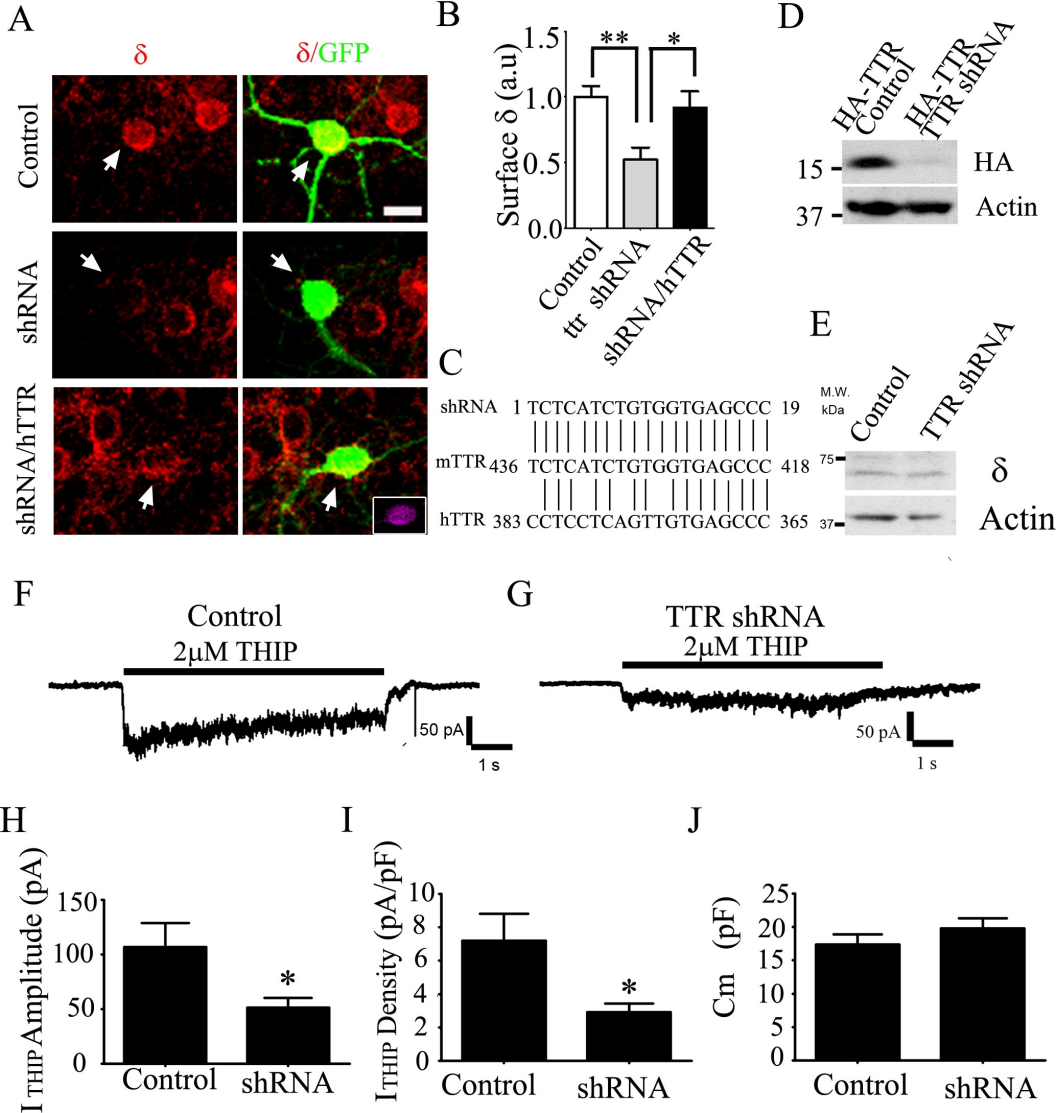
TTR regulates the surface expression of δ-receptors in cultured cerebellar neurons. (**A**) Knockdown TTR with mouse-specific shRNAs reduced the surface δ-staining (red), which was rescued by co-transection with human TTR (RFP-tagged, purple in the insert) that was resistant to mouse TTR shRNAs. Arrows point to the transfected cells. Scale bar, 10 μm. (**B**) Quantification of the surface δ staining signal shown in (**A**). * P < 0.05; ** P < 0.01. (**C**) Nucleotide sequence alignment showing the specificity of the TTR shRNA designed for mouse TTR, but not for human TTR. (**D**) Knockdown efficiency of the TTR shRNAs evaluated by cotransfecting mouse HA-TTR with control plasmid or with TTR specific shRNAs in HEK cells. TTR shRNAs significantly downregulated the expression level of mouse HA-TTR. (**E**) TTR shRNAs had no off-target effect directly on δ-GABA_A_-Rs when coexpressed in HEK cells. (**F-G**) Representative traces of THIP current recorded from cultured cerebellar granule cells either transfected with GFP as a control (**F**) or with TTR shRNA (**G**). (**H-J**) Summarized bar graphs showing the THIP current amplitude (**H**; Control: 107 ± 25 pA, n = 18; TTR shRNA: 55 ± 12 pA, n = 22; P < 0.05, Students’ *t*-test.), THIP current density (**I**; Control: 7.2 ± 1.7 pA/pF, n = 18; TTR shRNA: 2.9 ± 0.6 pA/pF, n = 22; P < 0.05), and membrane capacitance (**J**; Control: 17.4 ± 4.1 pF, n = 18; TTR shRNA: 19.8 ± 4.2 pF, n = 22; P > 0.2) changes after transfection of TTR shRNAs in cultured cerebellar granule cells. * P < 0.05.

To investigate whether TTR directly regulates the δ-GABA_A_-R expression in neuronal cells, we knocked down TTR expression with mouse-specific shRNAs in cultured cerebellar granule cells. As shown in Fig 3A, knockdown of TTR resulted in a significant reduction of the surface δ expression level. Importantly, human TTR, which is resistant to the mouse shRNAs (see sequence alignment in Fig 3C), could rescue the deficit of the surface δ staining (Fig 3B; Control, 1 ± 0.08, n = 22; TTR shRNA, 0.52 ± 0.093, n = 34; TTR shRNA + hTTR, 0.92 ± 0.12, n = 19). The knockdown efficiency of TTR shRNA on mouse TTR was demonstrated in Fig 3D (75% reduction in the presence of shRNA; p < 0.03). We also demonstrated that TTR shRNA had no off-target effect on the δ subunit (Fig 3E).

To further understand the functional regulation of TTR on the extrasynaptic δ-GABA_A_-Rs, we employed electrophysiological recordings to measure δ-GABA_A_-R mediated tonic currents in cerebellar granule cell cultures. Low concentration of THIP is a relatively specific agonist for δ-GABA_A_-Rs [13]. We found that application of THIP (2 μM) induced a significant tonic current in cerebellar granule cells (Fig 3F), indicating the presence of δ-GABA_A_-Rs as revealed by surface immunostaining. Importantly, the tonic current was significantly reduced after knocking down TTR in cerebellar granule neurons, consistent with the reduction of surface δ-staining induced by TTR shRNA (Fig 3G). Quantification indicated that both the THIP current amplitude and the current density were significantly reduced in TTR knockdown cells (Fig 3H and 3I; p < 0.05), whereas the capacitance of cells, a measure of cell size was not changed (Fig 3J).

**Fig 4:**
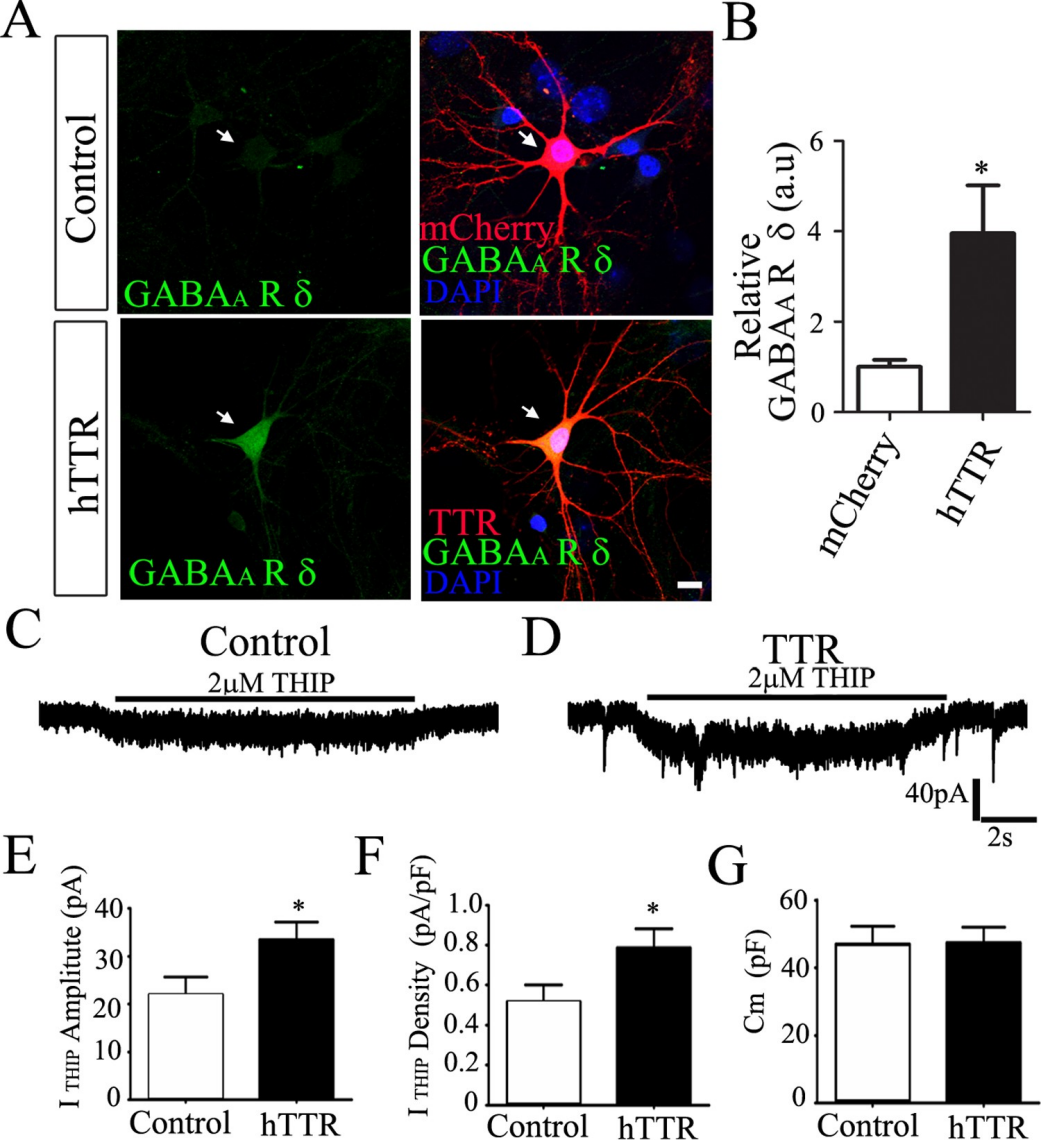
Overexpression of TTR in cultured cortical neurons increased the surface δ expression and tonic current. **(A**) Overexpression of human TTR increased the surface δ signal in cortical neurons. Arrows point to the cells transfected with mCherry (control) or TTR. Scale bar, 10 μm. (**B**) Quantification of the surface δ signal intensity in control and TTR-overexpressing cells. * P < 0.04. (**C-D**) Representative traces of THIP currents recorded from cortical neurons transfected with mCherry (**C**, control) or human TTR (**D**). (**E-G**) Summary bar graphs for THIP current amplitude (**E**; Control: 22 ± 3 pA, n = 17; TTR: 33 ± 3 pA, n = 17; * P < 0.05, Student’s *t*-test), THIP current density (**F;** Control: 0.52 ± 0.08 pA/pF, n = 17; TTR: 0.79 ± 0.09 pA/pF, n = 17; * P < 0.05), and cell membrane capacitance (**G;** Control: 47 ± 5 pF, n = 17; TTR: 47 ± 4 pF, n = 17; P > 0.9).

We next examined whether increasing intracellular synthesis of TTR will affect the expression of the δ-GABA_A_-Rs. For this purpose, we tested in cultured cortical neurons, which normally do not express δ-GABA_A_-Rs. As shown in the control cortical neurons expressing mCherry alone, the immunostaining of the δ subunit was essentially absent (Fig 4A, top row). In contrast, when cortical neurons were transfected with human TTR, the δ immunostaining signal was readily detected (Fig 4A, bottom row). Quantitative data showed a remarkable increase of the δ expression level after overexpressing hTTR in cortical neurons (Fig 4B; p < 0.05). These immunostaining results were further confirmed with electrophysiological analyses. To assess the level of the δ-GABA_A_-Rs in cortical neurons, we applied their specific agonist THIP (2 μM) to examine the activated tonic current. We found that THIP-induced tonic current was significantly increased in cortical neurons after overexpressing hTTR (Fig 4C–4G; p < 0.05). Together, our results demonstrated that TTR is a potent regulator of the δ-GABA_A_-Rs.

### Monomeric TTR not effective in regulating δ-GABA_A_-Rs

**Fig 5:**
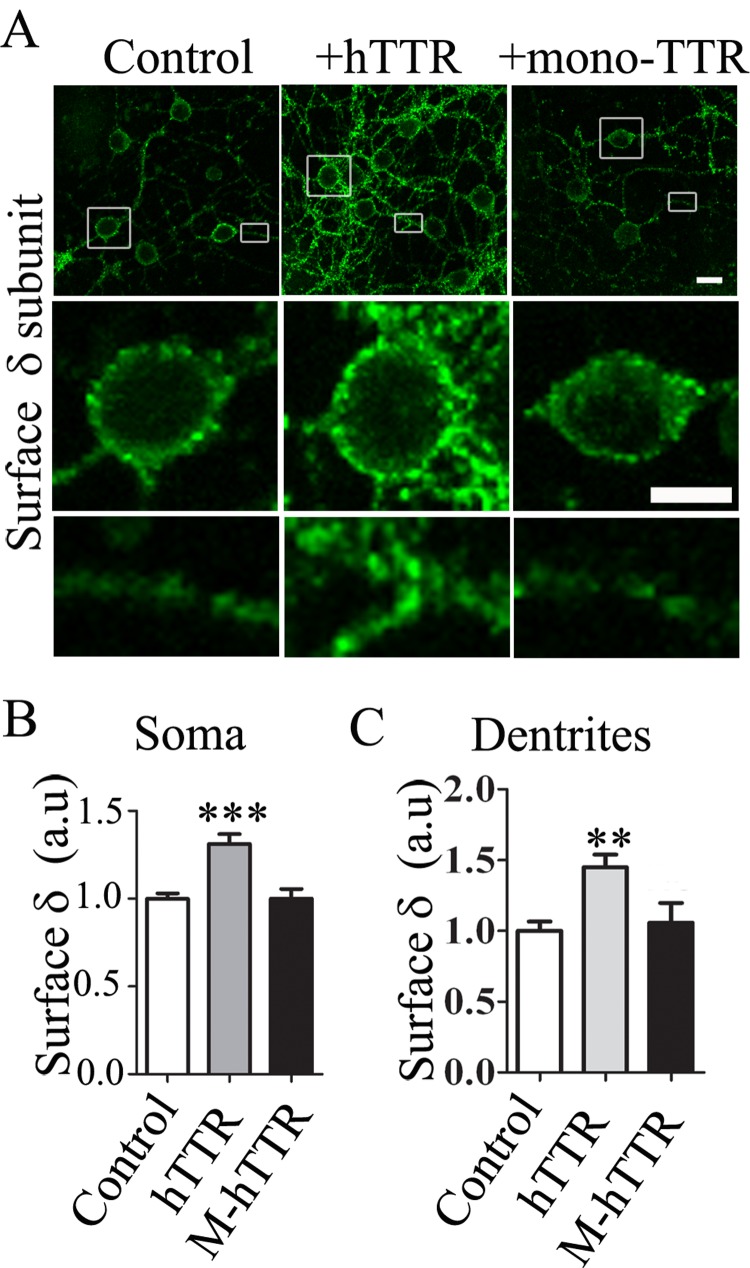
Effects of wide type human TTR peptides or engineered monomeric human TTR (M-hTTR) on δ-GABA_A_-Rs in cerebellar granule cells. (**A**) Immunostaining of surface δ subunit (green) in cerebellar granule neurons after treatment with 14 ug/ml hTTR or M-hTTR for 1 day. Scale bar, 5 μm and 2.5 μm. (**B-C**) Quantification of the surface δ signal on soma (**B**) or dendrites (**C**). *** P < 0.001, ** P < 0.01.

TTR is mostly synthesized in the liver and in choroid plexus in the brain[18]. Therefore, neurons are often exposed to TTR in the extracellular space. TTR normally circulates as a non-covalently bound homo-tetramer [34]. A mutant TTR has been engineered that cannot form tetramers and exists as a monomer [35]. To investigate which type of TTR, tetramer or monomer, regulates the δ-GABA_A_-Rs, we added either normal purified human TTR (hTTR) protein or purified monomeric hTTR (M-hTTR; both at 14 ug/ml) protein into the culture medium for 24 hrs before analyzing the surface level of δ-GABA_A_-Rs in cerebellar granule neurons. Both types of hTTR contain no tags. We found that normal hTTR protein significantly increased the expression level of δ-GABA_A_-Rs as expected, but monomeric hTTR had no effect on the δ-GABA_A_-Rs (Fig 5A). Quantitative analysis demonstrated that normal hTTR, but not monomeric hTTR, increased the surface expression of δ-GABA_A_-Rs in both soma and dendrites (Fig 5B; p < 0.01).

### *In vivo* analysis of TTR effect on δ-GABA_A_ receptors

**Fig 6:**
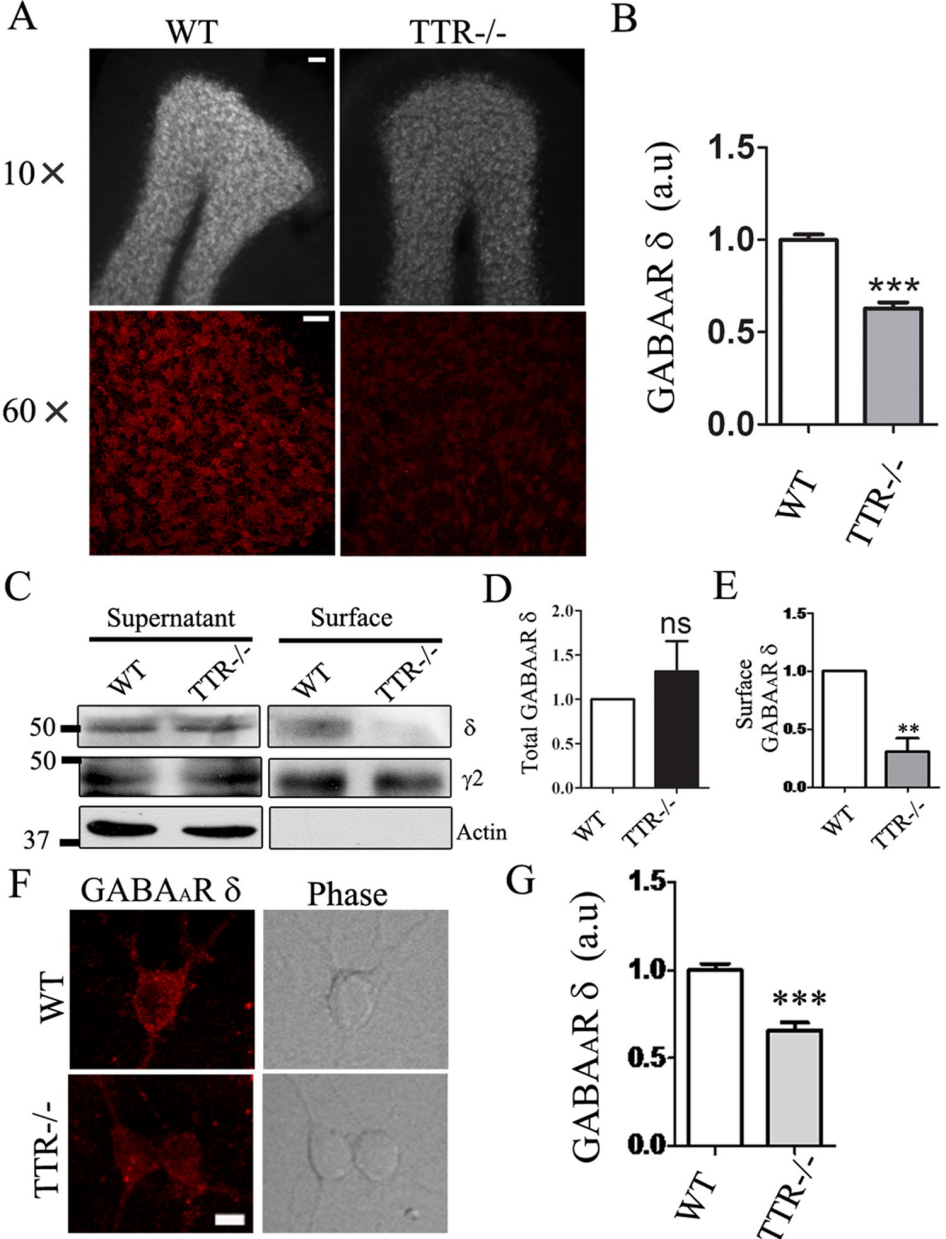
In vivo analysis of the δ-GABA_A_-Rs in the cerebellar granule layer in WT and TTR-/- mice. (**A**) Immunostaining of the surface δ receptors in cerebellar slices from WT and TTR-/- mice (7–8 months old). Top row, low power image of the δ signal in cerebellar granule layer. Scale bar, 50 μm. Bottom row, high power image of the δ signal. Scale bar, 20 μm. (**B**) Quantification of the relative surface δ signal density in WT and TTR-/- mice (low power image). (C) Western blot analysis of the total and surface δ receptors in WT and TTR-/- cerebellar tissue. The total δ protein level showed no difference, while the biotinylated surface δ protein level decreased significantly in TTR-/- mice. Both total and the surface γ2 GABA_A_-Rs have no difference between WT and TTR-/- mice. (D-E) Quantification of the relative total and surface δ intensity from the Western blot analysis. (F-G) Surface δ staining and quantification in primary cerebellar neuronal cultures from WT or TTR-/- mice. Bar, 5μm.

After *in vitro* analysis of TTR regulation of δ-GABA_A_-Rs in cell cultures, we further investigated TTR effect in mouse brain *in vivo* by using TTR knockout mice (TTR-/-) [36]. It is known that δ-subunit-containing GABAA receptors are expressed in cerebellar granule cells [29], so we immunostained the δ receptors in the cerebellum to examine TTR effect. We found that the δ signal was significantly reduced in the granule layer of TTR-/- mice (Fig 6A and 6B; p < 0.001). We further performed Western blot analysis for the total and surface δ receptors (biotinylated) in the cerebellar tissue. While the total δ receptor signal did not change, the surface δ receptor signal significantly reduced in the cerebellar granule layer of TTR-/- mice (Fig 6C–6E; p < 0.01). In contrast, no differences were found in both total and surface γ2 receptors between WT and TTR-/- mice (Fig 6C). Furthermore, we cultured cerebellar granule neurons from WT and TTR-/- mice and confirmed the surface staining of δ receptors significantly reduced in TTR-/- neurons (Fig 6F and 6G; p < 0.001). Thus, our *in vivo* analysis further demonstrated that TTR plays an important role in regulating the δ-GABA_A_-Rs.

## Discussion

The current study identified TTR as a novel interacting partner for the extrasynaptic δ subunit-containing GABA_A_-Rs. This was achieved by yeast two-hybrid screening of a mouse cerebellum cDNA library where the δ transcript is enriched. We demonstrated that TTR and the δ-GABA_A_-Rs can co-immunoprecipitate when coexpressed in HEK cells or directly lysed from mouse cerebellar tissue. Overexpression and knockdown experiments in cultured neurons suggest that TTR can potently regulate the expression level of the δ-GABA_A_-Rs. External application of normal TTR, which typically forms tetramers, or mutant monomeric TTR revealed that tetrameric TTR can regulate surface expression of δ-GABA_A_-Rs. In TTR-/- mice, we also observed a reduction of the δ-GABA_A_-R expression level in the cerebellar granule layer. Functionally, TTR regulates the tonic currents mediated by the δ-GABA_A_-Rs. Together our studies suggest that TTR is a critical regulator of the δ-GABA_A_-Rs.

### Identification of TTR as an interacting partner for the GABA_A_-R δ subunit

Using an unbiased yeast two-hybrid screening assay, we identified TTR as an interacting protein for the extracellular domain of the δ subunit of GABA_A_-Rs. We confirmed their interaction in HEK 293T cells through co-immunoprecipitation and co-localization experiments. Co-immunoprecipitation experiments from brain tissue lysates further demonstrated that TTR and the δ-GABA_A_-Rs interact with each other *in vivo*. In the brain, TTR is mainly secreted by choroid plexus into the cerebrospinal fluid [29]. Interestingly, cerebellum is in close proximity of one of the choroid plexuses in the brain, and thus may be under the influence of TTR secreted by the nearby choroid plexus. A recent study reported that sleep increases the exchange of cerebrospinal fluid with interstitial fluid in the brain [37]. Previous studies have already found that the δ-GABA_A_-Rs may play a role in sleep [38, 39]. Thus, our studies raised a possibility that TTR might be related to sleep by influencing the δ-GABA_A_-Rs.

### Regulation of δ-GABA_A_-Rs by TTR

Our intracellular manipulation of TTR expression in cultured neurons suggests that TTR is a potent regulator of δ-GABA_A_-Rs. We demonstrated that overexpression of TTR in cortical neurons, which typically have low expression of δ-GABA_A_-Rs, significantly increases the expression of δ-GABA_A_-Rs. Conversely, knockdown of TTR in cerebellar granule cells significantly downregulates the normally high expression level of δ-GABA_A_-Rs. The δ-GABA_A_-Rs are the major subtype of extrasynaptic GABA_A_-Rs and play an important role of tonic inhibition to regulate neuronal excitability in the brain [9]. Our recent work demonstrated that increasing extrasynaptic δ-GABA_A_-Rs in cortical neurons will result in a significant decrease of synaptic GABA transmission, suggesting a homeostatic competition between tonic and phasic GABA inhibition [3]. The potent regulation of TTR on the δ-GABA_A_-Rs suggests that TTR may play a role in modulating the tonic inhibition and thus tilting the balance between tonic and phasic inhibition. Indeed, we have demonstrated that overexpression of TTR can significantly increase tonic current, whereas knockdown of TTR can significantly decrease tonic current. Thus, besides its normal function of transporting thyroid hormone and retinol, our studies suggest that TTR may have a completely different function in regulating GABA inhibition in the brain.

Previous studies have reported that TTR also binds to amyloid β peptide (Aβ) and thus may play a role in Aβ clearance in Alzheimer’s disease brain [21, 22]. On the other hand, TTR itself may form aggregates and lead to systemic amyloidosis [40, 41]. Recent biochemical analysis suggested that in vivo it is likely the tetrameric TTR that binds to Aβ monomers and inhibits Aβ aggregation [22, 42]. This is consistent with our finding that TTR tetramer, not monomer, regulates the expression level of the δ-GABA_A_-Rs. Interestingly, TTR has been reported to be involved in cognition during aging [43] and associated with depression [44]. GABA_A_-Rs are known to play an important role in cognitive functions and are involved in mood disorders [7, 9, 45]. Whether the function of TTR in cognition and depression is mediated by the regulation of the δ-GABA_A_-Rs reported here will be an interesting topic to explore in future studies.

## References

[1] Jacob TC, Moss SJ, Jurd R. GABA(A) receptor trafficking and its role in the dynamic modulation of neuronal inhibition. Nat Rev Neurosci. 2008;9(5):331–43. Epub 2008/04/03. doi: nrn2370 [pii] 10.1038/nrn2370 18382465 PMC2709246

[2] Luscher B, Fuchs T, Kilpatrick CL. GABAA receptor trafficking-mediated plasticity of inhibitory synapses. Neuron. 2011;70(3):385–409. Epub 2011/05/11. doi: S0896-6273(11)00300-X [pii] 10.1016/j.neuron.2011.03.024 21555068 PMC3093971

[3] Wu X, Huang L, Wu Z, Zhang C, Jiang D, Bai Y, et al Homeostatic Competition between phasic and tonic inhibition. J Biol Chem. 2013;288:25053–65. Epub 2013/07/11. doi: M113.491464 [pii] 10.1074/jbc.M113.491464 . 23839941 PMC3757170

[4] Coghlan S, Horder J, Inkster B, Mendez MA, Murphy DG, Nutt DJ. GABA system dysfunction in autism and related disorders: from synapse to symptoms. Neurosci Biobehav Rev. 2012;36(9):2044–55. Epub 2012/07/31. doi: S0149-7634(12)00118-2 [pii] 10.1016/j.neubiorev.2012.07.005 . 22841562 PMC4477717

[5] Fritschy JM. Epilepsy, E/I Balance and GABA(A) Receptor Plasticity. Front Mol Neurosci. 2008;1:5 Epub 2008/10/24. 10.3389/neuro.02.005.2008 18946538 PMC2525999

[6] Lydiard RB. The role of GABA in anxiety disorders. J Clin Psychiatry. 2003;64 Suppl 3:21–7. Epub 2003/03/29. . 12662130

[7] Luscher B, Shen Q, Sahir N. The GABAergic deficit hypothesis of major depressive disorder. Mol Psychiatry. 2011;16(4):383–406. Epub 2010/11/17. doi: mp2010120 [pii] 10.1038/mp.2010.120 . 21079608 PMC3412149

[8] Lewis DA, Fish KN, Arion D, Gonzalez-Burgos G. Perisomatic inhibition and cortical circuit dysfunction in schizophrenia. Curr Opin Neurobiol. 2011;21(6):866–72. Epub 2011/06/18. doi: S0959-4388(11)00081-X [pii] 10.1016/j.conb.2011.05.013 21680173 PMC3183273

[9] Brickley SG, Mody I. Extrasynaptic GABA(A) receptors: their function in the CNS and implications for disease. Neuron. 2012;73(1):23–34. Epub 2012/01/17. doi: S0896-6273(11)01093-2 [pii] 10.1016/j.neuron.2011.12.012 . 22243744 PMC3399243

[10] Shen H, Sabaliauskas N, Sherpa A, Fenton AA, Stelzer A, Aoki C, et al A critical role for alpha4betadelta GABAA receptors in shaping learning deficits at puberty in mice. Science. 2010;327(5972):1515–8. Epub 2010/03/20. doi: 327/5972/1515 [pii] 10.1126/science.1184245 20299596 PMC2887350

[11] Maguire J, Mody I. GABA(A)R plasticity during pregnancy: relevance to postpartum depression. Neuron. 2008;59(2):207–13. Epub 2008/08/01. doi: S0896-6273(08)00537-0 [pii] 10.1016/j.neuron.2008.06.019 . 18667149 PMC2875248

[12] Nusser Z, Sieghart W, Somogyi P. Segregation of different GABAA receptors to synaptic and extrasynaptic membranes of cerebellar granule cells. J Neurosci. 1998;18(5):1693–703. . 9464994 10.1523/JNEUROSCI.18-05-01693.1998 PMC6792611

[13] Jia F, Pignataro L, Schofield CM, Yue M, Harrison NL, Goldstein PA. An extrasynaptic GABAA receptor mediates tonic inhibition in thalamic VB neurons. J Neurophysiol. 2005;94(6):4491–501. 10.1152/jn.00421.2005 . 16162835

[14] Smith SS, Shen H, Gong QH, Zhou X. Neurosteroid regulation of GABA(A) receptors: Focus on the alpha4 and delta subunits. Pharmacol Ther. 2007;116(1):58–76. Epub 2007/05/22. doi: S0163-7258(07)00070-8 [pii] 10.1016/j.pharmthera.2007.03.008 17512983 PMC2657726

[15] Farrant M, Nusser Z. Variations on an inhibitory theme: phasic and tonic activation of GABA(A) receptors. Nat Rev Neurosci. 2005;6(3):215–29. 10.1038/nrn1625 . 15738957

[16] Hamilton JA, Benson MD. Transthyretin: a review from a structural perspective. Cell Mol Life Sci. 2001;58(10):1491–521. 10.1007/PL00000791 . 11693529 PMC11337270

[17] Buxbaum JN, Reixach N. Transthyretin: the servant of many masters. Cell Mol Life Sci. 2009;66(19):3095–101. Epub 2009/08/01. 10.1007/s00018-009-0109-0 . 19644733 PMC4820353

[18] Soprano DR, Herbert J, Soprano KJ, Schon EA, Goodman DS. Demonstration of transthyretin mRNA in the brain and other extrahepatic tissues in the rat. J Biol Chem. 1985;260(21):11793–8. . 4044580

[19] Li X, Masliah E, Reixach N, Buxbaum JN. Neuronal production of transthyretin in human and murine Alzheimer's disease: is it protective? J Neurosci. 2011;31(35):12483–90. Epub 2011/09/02. doi: 31/35/12483 [pii] 10.1523/JNEUROSCI.2417-11.2011 21880910 PMC3172869

[20] Li X, Buxbaum JN. Transthyretin and the brain re-visited: is neuronal synthesis of transthyretin protective in Alzheimer's disease? Mol Neurodegener. 2011;6:79. Epub 2011/11/25. doi: 1750-1326-6-79 [pii] 10.1186/1750-1326-6-79 22112803 PMC3267701

[21] Buxbaum JN, Ye Z, Reixach N, Friske L, Levy C, Das P, et al Transthyretin protects Alzheimer's mice from the behavioral and biochemical effects of Abeta toxicity. Proc Natl Acad Sci U S A. 2008;105(7):2681–6. Epub 2008/02/15. doi: 0712197105 [pii] 10.1073/pnas.0712197105 18272491 PMC2268196

[22] Li X, Zhang X, Ladiwala AR, Du D, Yadav JK, Tessier PM, et al Mechanisms of transthyretin inhibition of beta-amyloid aggregation in vitro. J Neurosci. 2013;33(50):19423–33. Epub 2013/12/18. doi: 33/50/19423 [pii] 10.1523/JNEUROSCI.2561-13.2013 24336709 PMC3858619

[23] Silva CS, Eira J, Ribeiro CA, Oliveira A, Sousa MM, Cardoso I, et al Transthyretin neuroprotection in Alzheimer's disease is dependent on proteolysis. Neurobiol Aging. 2017;59:10–4. Epub 2017/08/07. 10.1016/j.neurobiolaging.2017.07.002 . 28780366

[24] Wu X, Wu Z, Ning G, Guo Y, Ali R, Macdonald RL, et al gamma-Aminobutyric Acid Type A (GABAA) Receptor alpha Subunits Play a Direct Role in Synaptic Versus Extrasynaptic Targeting. J Biol Chem. 2012;287(33):27417–30. Epub 2012/06/20. doi: M112.360461 [pii] 10.1074/jbc.M112.360461 . 22711532 PMC3431651

[25] Jiang M, Chen G. High Ca2+-phosphate transfection efficiency in low-density neuronal cultures. Nature Protocols. 2006;1(2):695–700. 10.1038/nprot.2006.86 17406298

[26] Deng L, Yao J, Fang C, Dong N, Luscher B, Chen G. Sequential postsynaptic maturation governs the temporal order of GABAergic and glutamatergic synaptogenesis in rat embryonic cultures. J Neurosci. 2007;27(40):10860–9. Epub 2007/10/05. doi: 27/40/10860 [pii] 10.1523/JNEUROSCI.2744-07.2007 . 17913919 PMC6672810

[27] Sun C, Zhang L, Chen G. An unexpected role of neuroligin-2 in regulating KCC2 and GABA functional switch. Mol Brain. 2013;6:23 Epub 2013/05/15. 10.1186/1756-6606-6-23 23663753 PMC3661362

[28] Kilpatrick CL, Murakami S, Feng M, Wu X, Lal R, Chen G, et al Dissociation of Golgi-associated DHHC-type Zinc Finger Protein (GODZ)- and Sertoli Cell Gene with a Zinc Finger Domain-beta (SERZ-beta)-mediated Palmitoylation by Loss of Function Analyses in Knock-out Mice. J Biol Chem. 2016;291(53):27371–86. Epub 2016/11/23. 10.1074/jbc.M116.732768 27875292 PMC5207163

[29] Schreiber G, Aldred AR, Jaworowski A, Nilsson C, Achen MG, Segal MB. Thyroxine transport from blood to brain via transthyretin synthesis in choroid plexus. Am J Physiol. 1990;258(2 Pt 2):R338–45. Epub 1990/02/01. 10.1152/ajpregu.1990.258.2.R338 . 2309926

[30] Cope DW, Hughes SW, Crunelli V. GABAA receptor-mediated tonic inhibition in thalamic neurons. J Neurosci. 2005;25(50):11553–63. Epub 2005/12/16. 10.1523/JNEUROSCI.3362-05.2005 . 16354913 PMC6726040

[31] Scimemi A, Andersson A, Heeroma JH, Strandberg J, Rydenhag B, McEvoy AW, et al Tonic GABA(A) receptor-mediated currents in human brain. Eur J Neurosci. 2006;24(4):1157–60. Epub 2006/08/26. 10.1111/j.1460-9568.2006.04989.x . 16930441

[32] Glykys J, Peng Z, Chandra D, Homanics GE, Houser CR, Mody I. A new naturally occurring GABA(A) receptor subunit partnership with high sensitivity to ethanol. Nat Neurosci. 2007;10(1):40–8. Epub 2006/12/13. 10.1038/nn1813 . 17159992

[33] Stell BM, Brickley SG, Tang CY, Farrant M, Mody I. Neuroactive steroids reduce neuronal excitability by selectively enhancing tonic inhibition mediated by delta subunit-containing GABAA receptors. Proc Natl Acad Sci U S A. 2003;100(24):14439–44. Epub 2003/11/19. 10.1073/pnas.2435457100 14623958 PMC283610

[34] Blake CC, Geisow MJ, Oatley SJ, Rerat B, Rerat C. Structure of prealbumin: secondary, tertiary and quaternary interactions determined by Fourier refinement at 1.8 A. J Mol Biol. 1978;121(3):339–56. Epub 1978/05/25. . 671542 10.1016/0022-2836(78)90368-6

[35] Jiang X, Smith CS, Petrassi HM, Hammarstrom P, White JT, Sacchettini JC, et al An engineered transthyretin monomer that is nonamyloidogenic, unless it is partially denatured. Biochemistry. 2001;40(38):11442–52. Epub 2001/09/19. . 11560492 10.1021/bi011194d

[36] Episkopou V, Maeda S, Nishiguchi S, Shimada K, Gaitanaris GA, Gottesman ME, et al Disruption of the transthyretin gene results in mice with depressed levels of plasma retinol and thyroid hormone. Proc Natl Acad Sci U S A. 1993;90(6):2375–9. Epub 1993/03/15. 8384721 10.1073/pnas.90.6.2375 PMC46089

[37] Xie L, Kang H, Xu Q, Chen MJ, Liao Y, Thiyagarajan M, et al Sleep drives metabolite clearance from the adult brain. Science. 2013;342(6156):373–7. Epub 2013/10/19. doi: 342/6156/373 [pii] 10.1126/science.1241224 24136970 PMC3880190

[38] Faulhaber J, Steiger A, Lancel M. The GABAA agonist THIP produces slow wave sleep and reduces spindling activity in NREM sleep in humans. Psychopharmacology (Berl). 1997;130(3):285–91. Epub 1997/04/01. . 9151364 10.1007/s002130050241

[39] Winsky-Sommerer R, Vyazovskiy VV, Homanics GE, Tobler I. The EEG effects of THIP (Gaboxadol) on sleep and waking are mediated by the GABA(A)delta-subunit-containing receptors. Eur J Neurosci. 2007;25(6):1893–9. Epub 2007/04/06. doi: EJN5455 [pii] 10.1111/j.1460-9568.2007.05455.x . 17408425

[40] Buxbaum J, Tagoe C, Gallo G, Reixach N, French D. The pathogenesis of transthyretin tissue deposition: lessons from transgenic mice. Amyloid. 2003;10 Suppl 1:2–6. Epub 2003/12/03. . 14640034

[41] Zhao L, Buxbaum JN, Reixach N. Age-related oxidative modifications of transthyretin modulate its amyloidogenicity. Biochemistry. 2013;52(11):1913–26. Epub 2013/02/19. 10.1021/bi301313b 23414091 PMC3604100

[42] Cascella R, Conti S, Mannini B, Li X, Buxbaum JN, Tiribilli B, et al Transthyretin suppresses the toxicity of oligomers formed by misfolded proteins in vitro. Biochim Biophys Acta. 2013;1832(12):2302–14. Epub 2013/10/01. 10.1016/j.bbadis.2013.09.011 . 24075940

[43] Brouillette J, Quirion R. Transthyretin: a key gene involved in the maintenance of memory capacities during aging. Neurobiol Aging. 2008;29(11):1721–32. 10.1016/j.neurobiolaging.2007.04.007 . 17512093

[44] Sousa JC, Grandela C, Fernandez-Ruiz J, de Miguel R, de Sousa L, Magalhaes AI, et al Transthyretin is involved in depression-like behaviour and exploratory activity. J Neurochem. 2004;88(5):1052–8. . 15009661 10.1046/j.1471-4159.2003.02309.x

[45] Braudeau J, Delatour B, Duchon A, Pereira PL, Dauphinot L, de Chaumont F, et al Specific targeting of the GABA-A receptor alpha5 subtype by a selective inverse agonist restores cognitive deficits in Down syndrome mice. J Psychopharmacol. 2011;25(8):1030–42. Epub 2011/06/23. doi: 0269881111405366 [pii] 10.1177/0269881111405366 21693554 PMC3160204

